# Incremental Prognostic Value of Cardiac MRI Feature Tracking and T1 Mapping in Arrhythmogenic Right Ventricular Cardiomyopathy

**DOI:** 10.1148/ryct.230430

**Published:** 2024-10-24

**Authors:** Guanyu Lu, Liqi Cao, Weitao Ye, Xiaoyu Wei, Jiajun Xie, Zhicheng Du, Xinyue Zhang, Xinyi Luo, Jiehao Ou, Qianhuan Zhang, Yang Liu, Yuelong Yang, Hui Liu

**Affiliations:** From the Department of Radiology (G.L., L.C., W.Y., X.L., J.O., Y.Y., H.L.) and Guangdong Cardiovascular Institute (Q.Z., Y.L.), Guangdong Provincial People’s Hospital (Guangdong Academy of Medical Sciences), Southern Medical University, No. 106 Zhongshan 2nd Road, Guangzhou 510080, China; Department of Interventional Diagnosis and Therapy, Beijing Anzhen Hospital, Capital Medical University, Beijing, China (G.L.); Department of Radiology, Sun Yat-sen Memorial Hospital, Sun Yat-sen University, Guangzhou, China (X.W.); Department of Radiology, Guangzhou First People’s Hospital, School of Medicine, South China University of Technology, Guangzhou, China (J.X.); Department of Medical Statistics, School of Public Health, Sun Yat-sen University, Guangzhou, China (Z.D.); Department of Pediatrics, The First Clinical College, Guangdong Medical University, Zhanjiang, China (X.Z.); and School of Medicine, South China University of Technology, Guangzhou, China (X.L., H.L.).

**Keywords:** Cardiovascular MRI, Feature Tracking, T1 Mapping, Arrhythmogenic Right Ventricular Cardiomyopathy, Sustained Ventricular Arrhythmias

## Abstract

**Purpose:**

To explore the role of cardiac MRI feature tracking (FT) and T1 mapping in predicting sustained ventricular arrhythmias (VA) in patients with arrhythmogenic right ventricular cardiomyopathy (ARVC) and to investigate their possible incremental value beyond ARVC risk score.

**Materials and Methods:**

The retrospective study analyzed 91 patients with ARVC (median age, 36 years [IQR, 27–50 years]; 60 male, 31 female) who underwent cardiac MRI examinations between November 2010 and March 2022. The primary end point was the first occurrence of sustained VA after cardiac MRI to first VA, with censoring of patients who were alive without VA at last follow-up. Cox regression analysis was performed to assess the association between variables and time to sustained VA. Time-dependent receiver operating characteristic (ROC) analysis was performed to determine the incremental value of cardiac MRI FT and T1 mapping.

**Results:**

During a median follow-up of 55.0 months (IQR, 37.0–76.0 months), 36 of 91 (40%) patients experienced sustained VA. A 1% worsening in left ventricular global longitudinal peak strain (GLS), 1% worsening in right ventricular GLS, and a 1% increase in extracellular volume fraction (ECV) were associated with increased risk of sustained VA, with hazard ratios of 1.14 (95% CI: 1.06, 1.23; *P* = .001), 1.09 (95% CI: 1.02, 1.16; *P* = .02), and 1.13 (95% CI: 1.08, 1.18; *P* < .001), respectively, after adjustment for ARVC risk score. Adding both biventricular GLS and ECV to ARVC risk score showed significant incremental value for predicting sustained VA (area under the ROC curve: 0.73 vs 0.65; *P* < .001).

**Conclusion:**

Cardiac MRI–derived biventricular GLS and ECV provided independent and incremental value for predicting sustained VA beyond ARVC risk score alone in patients with ARVC.

**Keywords:** Cardiovascular MRI, Feature Tracking, T1 Mapping, Arrhythmogenic Right Ventricular Cardiomyopathy, Sustained Ventricular Arrhythmias

*Supplemental material is available for this article*

Published under a CC BY 4.0 license

## Graphical abstract

**Figure:**
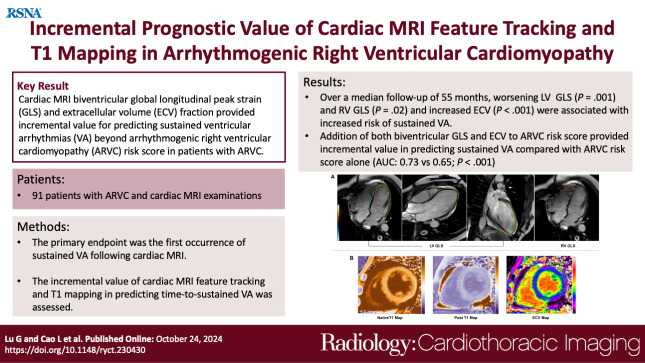

**Summary**

Cardiac MRI biventricular global longitudinal peak strain and extracellular volume fraction provided incremental value for predicting sustained ventricular arrhythmias beyond arrhythmogenic right ventricular cardiomyopathy (ARVC) risk score alone in patients with ARVC.

**Key Points**

■ During a median follow-up of 55.0 months, a 1% worsening in left ventricular global longitudinal peak strain (GLS) and right ventricular GLS and a 1% increase in extracellular volume fraction (ECV) were associated with a 14%, 9%, and 13% increase in the instantaneous risk of sustained ventricular arrhythmias (VA), respectively, after adjustment for the arrhythmogenic right ventricular cardiomyopathy (ARVC) risk score in patients with ARVC.
■ The addition of biventricular GLS (area under the receiver operating characteristic curve [AUC]: 0.70 vs 0.65; *P* = .02) or ECV (AUC: 0.70 vs 0.65; *P* = .002) to the ARVC risk score increased the capability for predicting the occurrence of sustained VA.
■ Combined assessment of cardiac MRI biventricular GLS and ECV provided incremental value for predicting VA beyond ARVC risk score alone (AUC: 0.73 vs 0.65; *P* < .001) in patients with ARVC.

## Introduction

Arrhythmogenic right ventricular cardiomyopathy (ARVC), an inherited cardiomyopathy, is pathologically characterized by progressive deletion of cardiomyocytes and fibrofatty replacement of the myocardium, leading to myocardial remodeling and dysfunction (1,2). It was initially described to predominantly involve the right ventricle, but recent studies have revealed biventricular or left-dominant forms of the disease (3,4). Ventricular arrhythmias (VA) and sudden cardiac death (SCD) may occur as the first but most serious clinical manifestation of ARVC. Therefore, accurate risk stratification of adverse cardiac events is critically important and may improve prognostication through timely implantable cardioverter-defibrillator (ICD) implantation (5).

Recently, Cadrin-Tourigny et al (6) proposed a novel ARVC risk score model for VA risk stratification in ARVC that was validated in external independent cohorts. However, the model seemed to underestimate the risk of VA in ARVC with left ventricular (LV) involvement. Furthermore, its predictive power, with a C statistic of 0.7, leaves room for improvement. Additional imaging biomarkers may improve prediction performance (7).

Cardiac MRI plays a key role in the evaluation of biventricular myocardial function and noninvasive tissue characterization in ARVC (1). However, cardiac MRI parameters other than right ventricular (RV) ejection fraction (RVEF) were not included in the final model. Cardiac MRI feature tracking (FT), a reliable technique with excellent reproducibility for accurately quantifying myocardial deformation in different orientations (8–11), can depict functional changes preceding a decrease in the ejection fraction (12). Although the wide prognostic implications of cardiac MRI FT in various cardiovascular conditions have been certified (12–14), its prognostic role in ARVC remains controversial and requires further clarification (15,16). The T1 mapping technique provides a noninvasive and reproducible method to detect diffuse myocardial fibrosis, which may constitute a substrate for the re-entrant mechanism of VA in ARVC (17,18). Nonetheless, evidence regarding the prognostic role of T1 mapping in ARVC is limited (3). Moreover, whether cardiac MRI FT and T1 mapping, as sensitive and early markers of adverse cardiac events (12,19), can provide additional predictive value over the ARVC risk score model remains unreported.

Therefore, the aim of the present study was to explore the role of cardiac MRI FT and T1 mapping in predicting sustained VA and to investigate their incremental predictive value beyond the ARVC risk score in patients with ARVC.

## Materials and Methods

This retrospective study was approved by the institutional review board of Guangdong Provincial People’s Hospital, and the requirement for informed consent was waived.

### Study Sample

**Figure 1:**
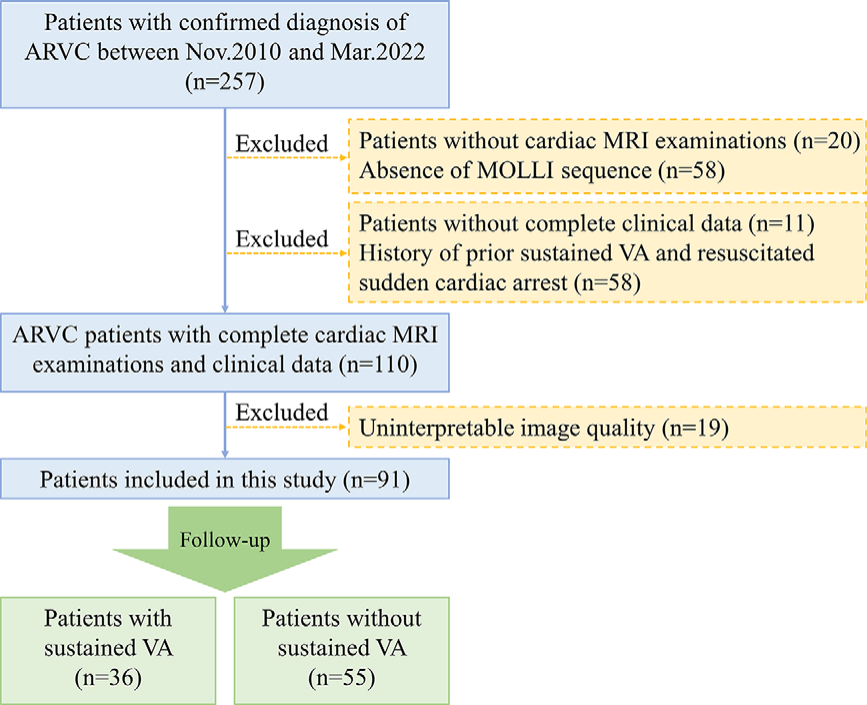
Flowchart of patient inclusion and exclusion. ARVC = arrhythmogenic right ventricular cardiomyopathy, MOLLI = modified Look-Locker inversion recovery, VA = ventricular arrhythmias.

A total of 257 patients were diagnosed with ARVC at our institution between November 2010 and March 2022. We established a diagnosis of ARVC on the basis of the revised 2010 Task Force Criteria. All patients included in the study met at least two major or one major plus two minor criteria or four minor criteria from different categories (2). Furthermore, according to the Padua criteria (20), LV involvement was considered if one or more of the following criteria were present: left ventricular ejection fraction (LVEF) less than 50%, LV wall motion abnormalities (hypokinesia, akinesia, dyskinesia), or LV late gadolinium enhancement (LGE) or fibrosis (stria or band pattern) affecting more than one segment based on the American Heart Association 17-segment model. Exclusion criteria were as follows: patients without cardiac MRI examinations (*n* = 20), absence of the modified Look-Locker inversion recovery sequence (*n* = 58) (detailed in Appendix S1), patients without complete clinical data (*n* = 11), any history of sustained VA or resuscitated sudden cardiac arrest before cardiac MRI examination (*n* = 58), and uninterpretable image quality (lack of image registration between pre- and postcontrast T1 mapping [*n* = 7], respiratory motion artifacts [*n* = 5], cardiac motion artifacts [*n* = 4], arrhythmia artifacts [*n* = 3]) (Fig 1). Patients with conditions potentially causing significant RV and/or LV remodeling were also carefully excluded. Forty healthy volunteers with a similar age and sex distribution served as controls. The healthy controls, with normal cardiac MRI findings, had no history of cardiovascular or metabolic disease.

### Cardiac MRI Protocol

Cardiac MRI examinations were performed using a 3.0-T scanner (Ingenia; Philips Medical Systems) according to the recommended cardiac MRI protocol (21). The imaging protocol included steady-state free precession cine imaging, T2-weighted imaging, LGE, and modified Look-Locker inversion recovery T1 mapping. The detailed protocol and parameters of sequences are shown in the Cardiac MRI Protocol section of Appendix S1.

### Cardiac MRI Analyses

**Figure 2:**
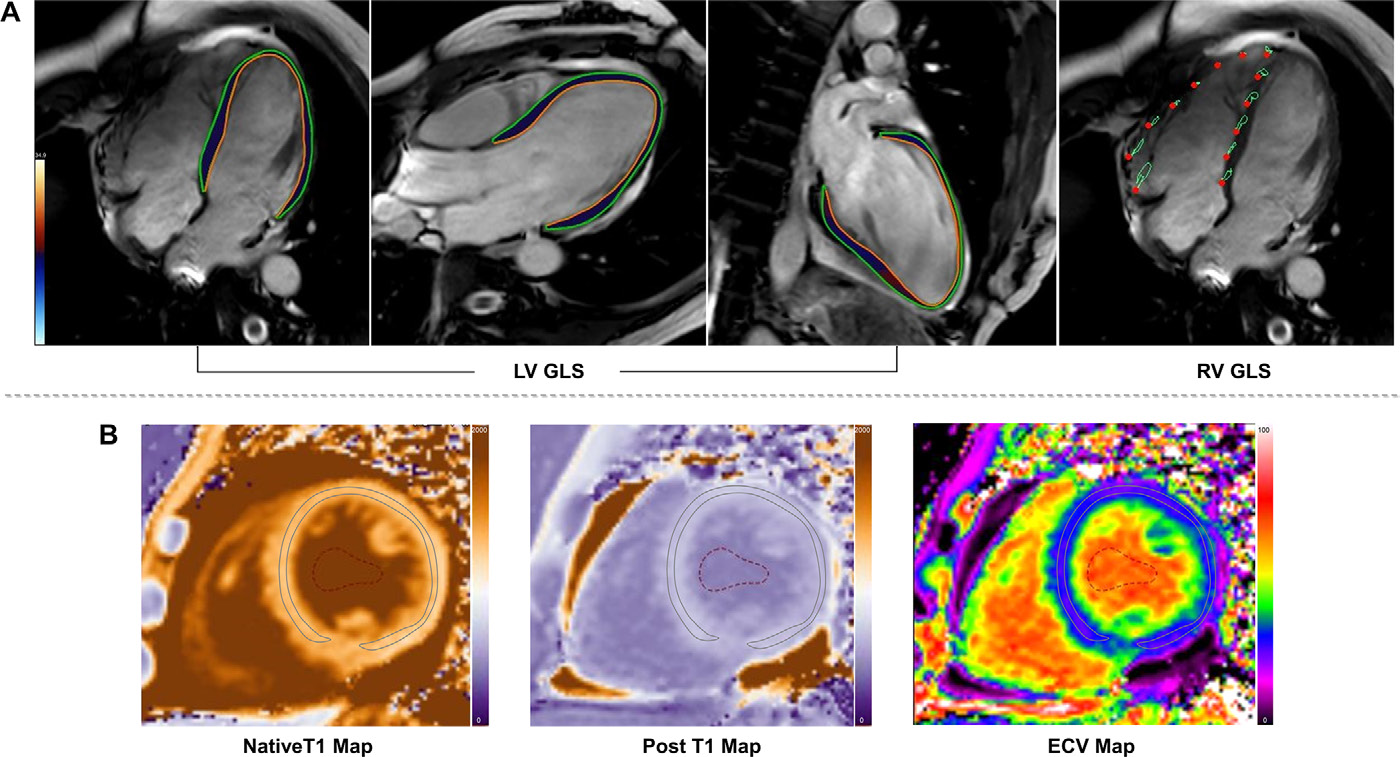
Images of cardiac MRI biventricular global longitudinal peak strain (GLS) analysis and extracellular volume fraction (ECV) map. A 42-year-old female patient diagnosed with arrhythmogenic right ventricular cardiomyopathy based on the revised 2010 Task Force criteria who experienced sustained ventricular arrhythmias (VA) as a clinical end point during follow-up. **(A)** Left ventricular (LV) GLS (−12.97%) in long-axis planes (four-, three-, two-chamber view) and right ventricular (RV) GLS (−11.16%) in long-axis plane (four-chamber view). **(B)** Native T1, post-T1, and ECV (31.2%) map in the midventricular section of short-axis plane.

Cardiac MRI analyses were performed using commercially available postprocessing software (version 2.0, Medis). The detailed process and parameters for cardiac structural and functional evaluation are presented in the Cardiac MRI Analysis section of Appendix S1 (Fig 2).

### Outcomes and Follow-up

The collection of follow-up data and adjudication of clinical outcomes were conducted by two experienced cardiologists (Q.Z. and Y.L., with 20 and 18 years of experience in diagnosing and treating cardiovascular diseases, respectively). Follow-up data were obtained by reviewing medical records, clinical evaluations, and patient interviews. Clinical outcomes were adjudicated via review of electrocardiographic tracings, ICD interrogation tracings, and medical and death records. The primary end point was the occurrence of sustained VA during follow-up, with time to event defined as the period from the date of cardiac MRI to the date of the first occurrence of sustained VA, with censoring of patients who were alive without VA at the latest follow-up date. Sustained VA was defined as spontaneous sustained ventricular tachycardia lasting 30 seconds or longer at a heart rate of 100 beats per minute or greater, ventricular fibrillation, SCD or SCD abortion, and appropriate ICD intervention (6). An appropriate ICD intervention was designated as ICD shock therapy or antitachycardia pacing therapy in response to life-threatening arrhythmias, such as fast ventricular fibrillation or sustained ventricular tachycardia.

### Statistical Analysis

Continuous variables were presented as means ± SDs or medians (IQRs) and were compared using an independent Student *t* test or Mann–Whitney *U* test. Categorical variables were presented as frequencies (percentages) and were compared using the χ^2^ test or Fisher exact test, as appropriate.

Cox proportional hazard regression analyses were performed to assess the association between variables and time to sustained VA. To investigate the incremental role of cardiac MRI FT and T1 mapping in predicting time to sustained VA, four models were created. The ARVC risk score (model 1) served as the baseline model. The ARVC risk score was calculated using an online calculator available at *http://www.arvcrisk.com*, based on such factors as male sex, age, recent cardiac syncope, previous nonsustained ventricular tachycardia, 24-hour premature ventricular contraction count, number of leads with T-wave inversion, and RVEF (2). Cardiac MRI FT and T1 mapping parameters with a *P* value less than .05 on univariable screening were added sequentially in the multivariable analysis, resulting in model 2: model 1 plus biventricular global longitudinal peak strain (GLS); model 3: model 1 plus extracellular volume fraction (ECV); and model 4: model 1 plus biventricular GLS and ECV. Given the limited number of events (<40) and to avoid overfitting, several additional multivariable models were performed, incorporating LV involvement or LV LGE with biventricular GLS and ECV, as well as ARVC risk score with LV LGE. The proportional hazard assumption was evaluated using the Schoenfeld residuals test. Time-dependent receiver operating characteristic (ROC) analyses were performed to evaluate the abilities of models in predicting sustained VA at 5 years, comparing the areas under the ROC curves (AUCs) of models with and without GLS and ECV.

Kaplan–Meier plots for biventricular GLS and ECV stratified by their median values were used to describe event-free survival for sustained VA and were analyzed using log-rank tests. Kaplan–Meier plots for ARVC risk score were stratified into distinct groups (≤5%, >5% to ≤15%, >15% to ≤25%, >25% to ≤50%, and >50%).

Blinded to clinical characteristics and outcomes, observers analyzed 20 patients randomly selected from the entire cohort to assess the intra- and interobserver reproducibility of the cardiac MRI FT measurement using the intraclass correlation coefficient (ICC) and Bland–Altman analysis.

A two-sided *P* value less than .05 was considered to represent a statistically significant difference. Statistical analyses were performed using SPSS software, version 26 (IBM); MedCalc statistical software; GraphPad Prism 9.0 software (GraphPad Software); and R software, version 4.3.3 (R Foundation for Statistical Computing).

## Results

### Study Sample Characteristics

A total of 91 patients with ARVC (median age, 36 years [IQR, 27–50 years]; 60 male, 31 female) were included in the analysis (Fig 1). The major clinical manifestations of the patients were nonsustained ventricular tachycardia (61 of 91 [67%]), 24-hour premature ventricular complex count of 1000 or greater (41 of 91 [45%]), and cardiac syncope (32 of 91 [35%]). Of the 56 patients who underwent genetic testing, 40 tested positive for a genetic mutation, and 16 tested negative. Among the 40 patients with a positive genetic mutation, *PKP2* mutation (*n* = 20) was most commonly observed, followed by *DSP* (*n* = 10). The median ARVC risk score was 28.5% (IQR, 10.6%–48.7%) (Table 1). Baseline cardiac MRI characteristics are presented in Table 2. LV GLS and RV GLS were significantly impaired in patients with ARVC compared with healthy controls (*P* < .05) (Tables S1, S2). The native T1 value and ECV were significantly higher in patients with ARVC compared with healthy controls (*P* < .05) (Table S1). Overall, 63 of 91 (69%) patients exhibited LV involvement.

**Table 1:**
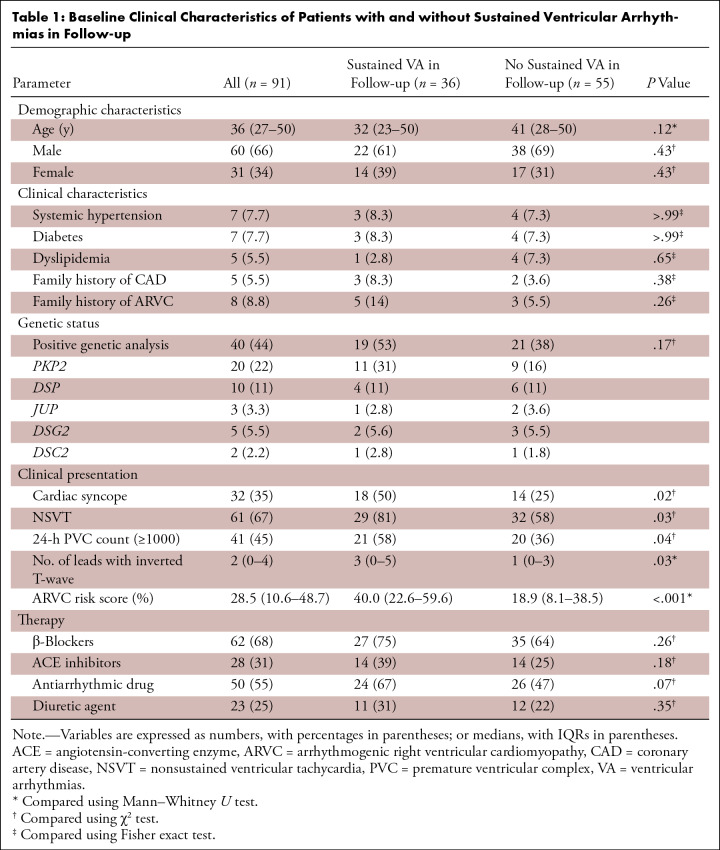
Baseline Clinical Characteristics of Patients with and without Sustained Ventricular Arrhythmias in Follow-up

**Table 2:**
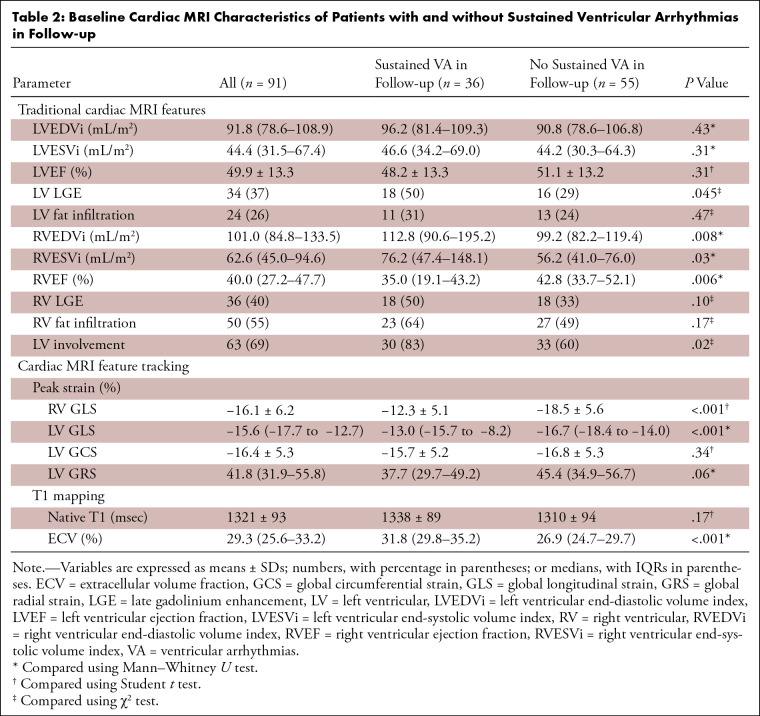
Baseline Cardiac MRI Characteristics of Patients with and without Sustained Ventricular Arrhythmias in Follow-up

### Clinical Characteristics Based on the Primary End Point

During a median follow-up period of 55.0 months (IQR, 37.0–76.0 months), 59 of 91 (65%) patients underwent ICD implantation. Thirty-six of 91 (40%) patients experienced sustained VA, including appropriate ICD intervention (*n* = 30), spontaneous sustained ventricular tachycardia (*n* = 2), and SCD (*n* = 4). Compared with those without sustained VA, patients who experienced sustained VA during follow-up exhibited a greater frequency of cardiac syncope (18 of 36 [50%] vs 14 of 55 [25%]; *P* = .02) and nonsustained ventricular tachycardia (29 of 36 [81%] vs 32 of 55 [58%]; *P* = .03). Patients who experienced sustained VA during follow-up had higher ARVC risk score than those free from sustained VA (40.0% [IQR, 22.6%–59.6%] vs 18.9% [IQR, 8.1%–38.5%]; *P* = .001) (Table 1).

### Cardiac MRI Characteristics Based on the Primary End Point

Patients with ARVC with the primary end point of sustained VA following cardiac MRI examination had higher RV end-diastolic and end-systolic volume index (*P* < .05) and lower RVEF (35.0% [IQR, 19.1%–43.2%] vs 42.8% [IQR, 33.7%–52.1%]; *P* = .006) compared with those without. No evidence of differences was observed in LV volume and function. LV LGE was present more often in patients who experienced sustained VA during follow-up (18 of 36 [50%] vs 16 of 55 [29%]; *P* = .045), whereas there was no evidence of a difference in RV LGE, RV fat infiltration, and LV fat infiltration between the two groups. Regarding cardiac MRI FT parameters, patients who experienced sustained VA during follow-up had significantly impaired RV GLS (−12.3% ± 5.1 vs −18.5% ± 5.6; *P* < .001) and LV GLS (−13.0% [IQR, −15.7% to −8.2%] vs −16.7% [IQR, −18.4% to −14.0%]; *P* < .001) compared with those free from sustained VA. No evidence of differences was found in LV global circumferential peak strain and LV global radial peak strain between the two groups. Regarding the parameters of T1 mapping, ECV were significantly higher in patients with sustained VA during follow-up than in those without (31.8% [IQR, 29.8%–35.2%] vs 26.9% [IQR, 24.7%–29.7%]; *P* < .001). There was no evidence of a difference in native T1 values between the two groups (*P* = .09).

### Univariable and Multivariable Cox Regression Analyses

The univariable predictors of sustained VA are listed in Table 3. The ARVC risk score, LV LGE, LV involvement, LV GLS, RV GLS, and ECV were significantly associated with the risk of sustained VA in univariable analyses.

**Table 3:**
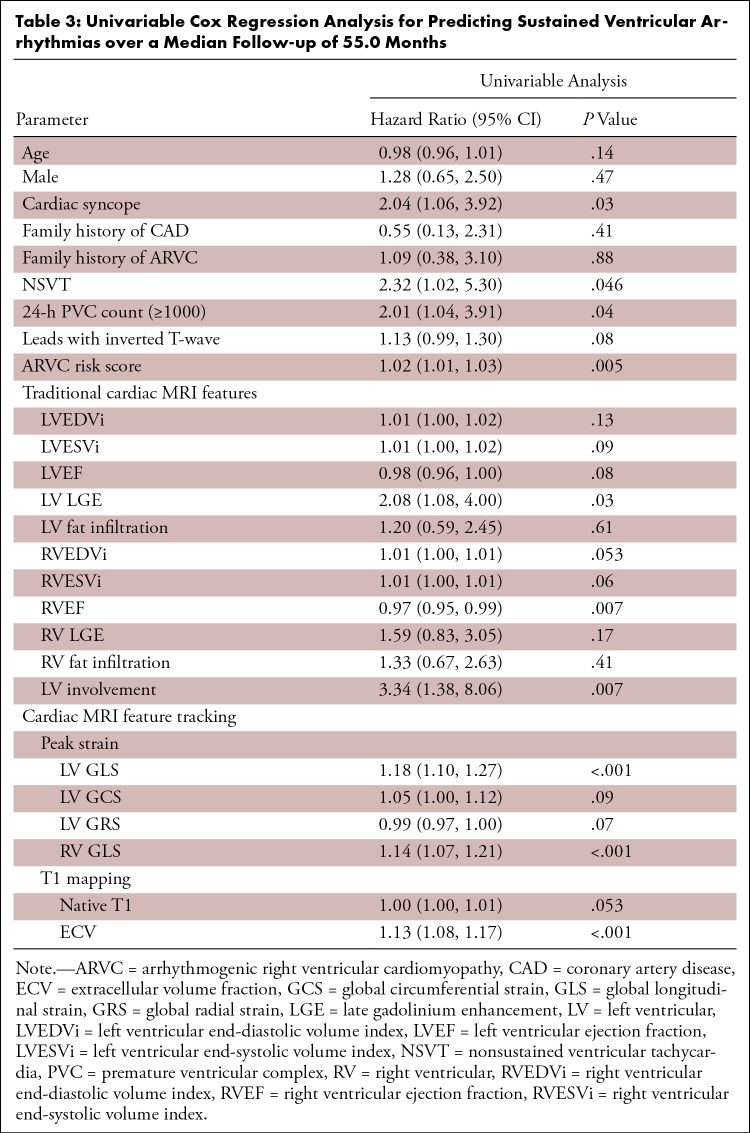
Univariable Cox Regression Analysis for Predicting Sustained Ventricular Arrhythmias over a Median Follow-up of 55.0 Months

In multivariable analyses, biventricular GLS and ECV separately maintained a significant association with the risk of sustained VA (Tables 4 and S3). During a median follow-up of 55.0 months, a 1% worsening in LV GLS (hazard ratio: 1.14 [95% CI: 1.06, 1.23]; *P* = .001) and a 1% worsening in RV GLS (hazard ratio: 1.09 [95% CI: 1.02, 1.16]; *P* = .02) were associated with a 14% and 9% increase in the instantaneous risk of sustained VA, respectively, after adjustment for the ARVC risk score. In addition, each 1% increase in ECV was associated with a 13% increased risk of sustained VA (hazard ratio: 1.13 [95% CI: 1.08, 1.18]; *P* < .001) after adjustment for the ARVC risk score (Table 4). Moreover, after separate adjustment for LV involvement and LGE, biventricular GLS and ECV remained independently associated with the risk of sustained VA (Tables S4, S5). LV LGE did not remain associated with the risk of VA after adjustment for ARVC risk score (Table S6). There was no evidence that the proportional hazard assumption was violated for any of the multivariable models (all global *P* > .05).

**Table 4:**
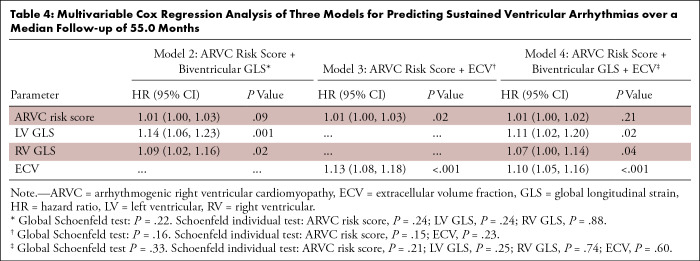
Multivariable Cox Regression Analysis of Three Models for Predicting Sustained Ventricular Arrhythmias over a Median Follow-up of 55.0 Months

**Figure 3:**
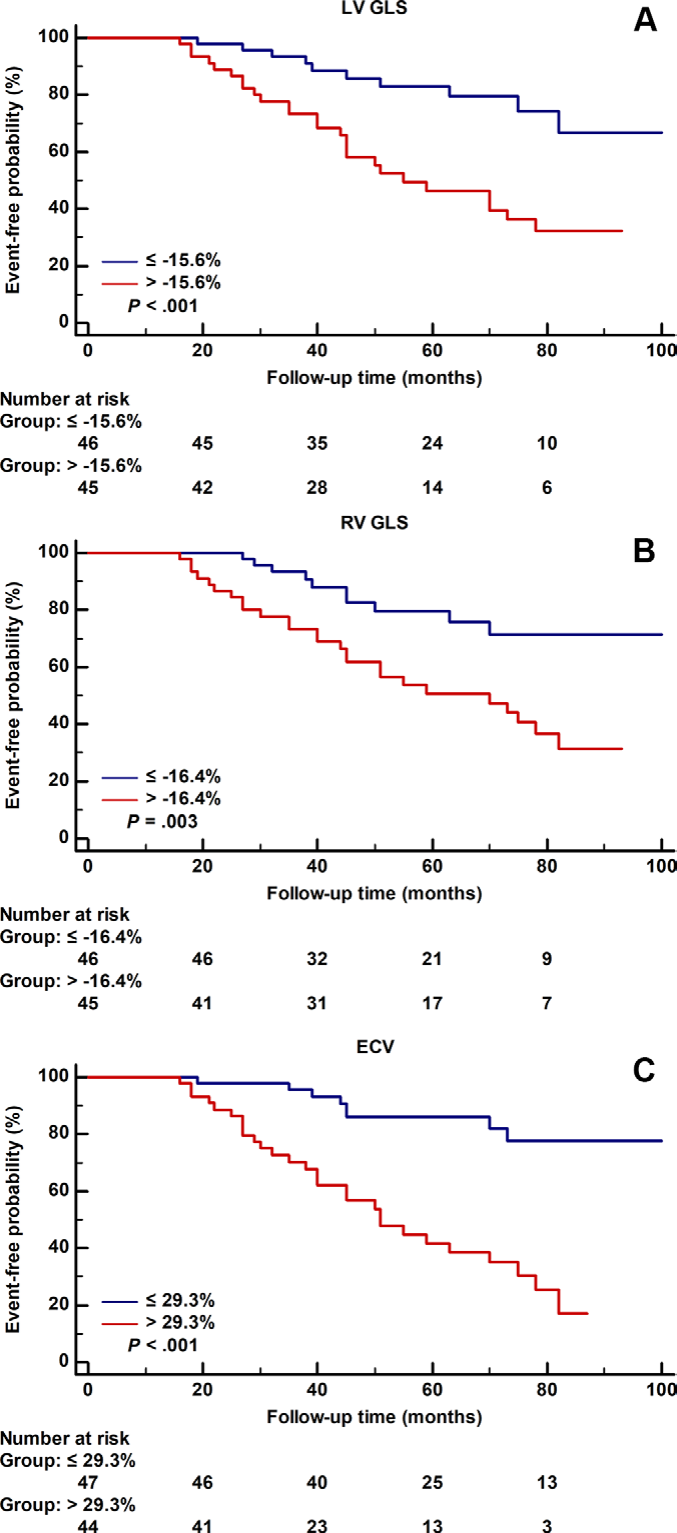
Event-free survival of patients with arrhythmogenic right ventricular cardiomyopathy stratified by global longitudinal peak strain (GLS) and extracellular volume fraction (ECV). Kaplan–Meier event-free probability curves for sustained ventricular arrhythmias according to the median values for left ventricular (LV) GLS, right ventricular (RV) GLS, and ECV. Patients with **(A)** LV GLS greater than −15.6%, **(B)** RV GLS greater than −16.4%, and **(C)** ECV greater than 29.3% experienced a significantly higher rate of sustained VA (all *P* < .05).

In addition, the risk of sustained VA increased with higher ARVC risk score (*P* = .02) (Fig S1). In Kaplan–Meier analysis stratified by their median values, there was an increased risk of sustained VA for patients exhibiting LV GLS greater than −15.6%, RV GLS greater than −16.4%, and ECV greater than 29.3% (all *P* < .05) (Fig 3).

### Incremental Value of Biventricular GLS and ECV

**Figure 4:**
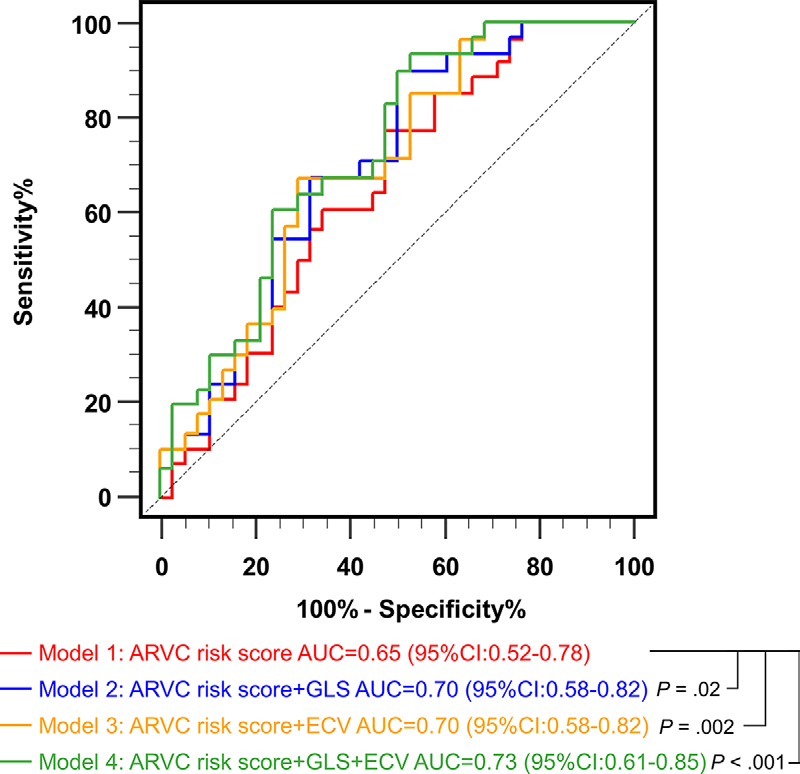
Receiver operating characteristic curves for predicting sustained ventricular arrhythmias at 5 years: incremental value of biventricular global longitudinal peak strain (GLS) and extracellular volume fraction (ECV) over arrhythmogenic right ventricular cardiomyopathy (ARVC) risk score. AUC = area under the receiver operating characteristic curve.

We further evaluated the abilities of the models before and after the addition of biventricular GLS and ECV in predicting sustained VA at 5 years. The addition of biventricular GLS (AUC, 0.70 [95% CI: 0.58, 0.82]; *P* = .02) or ECV (AUC, 0.70 [95% CI: 0.58, 0.82]; *P* = .002) improved the AUC for predicting sustained VA at 5 years compared with the baseline model (ARVC risk score) (AUC, 0.65 [95% CI: 0.52, 0.78]) (Figs 4 and S2). After both biventricular GLS and ECV were simultaneously added to the baseline model, the AUC increased and peaked (AUC, 0.73 [95% CI: 0.61, 0.85]; *P* < .001). We also found significant incremental values of biventricular GLS and ECV over LV involvement (*P* = .03) and LV LGE (*P* = .002) (Figs S3 and S4). However, LV LGE did not significantly add to the AUC of ARVC risk score (*P* = .72) (Fig S5).

### Reproducibility of Biventricular Global Strain Derived from Cardiac MRI FT

The ICCs for intra- and interobserver measurements of biventricular GLS derived from cardiac MRI FT are summarized in Table S7. All cardiac MRI FT parameters showed excellent reproducibility (intraobserver ICC, 0.905–0.941; interobserver ICC, 0.886–0.909). Bland–Altman plots visually revealed acceptable agreement for cardiac MRI FT at both the intra- and interobserver levels (Fig S6).

## Discussion

In this study, we explored the potential prognostic value of cardiac MRI FT and T1 mapping in ARVC. The principal findings were as follows: (*a*) During a median follow-up of 55.0 months, the hazard ratios for the association of sustained VA with a 1% worsening in LV GLS, a 1% worsening in RV GLS, and a 1% increase in ECV were 1.14 (95% CI: 1.06, 1.23; *P* = .001), 1.09 (95% CI: 1.02, 1.16; *P* = .02), and 1.13 (95% CI: 1.08, 1.18; *P* < .001), respectively, after adjustment for the ARVC risk score; (*b*) the addition of biventricular GLS (AUC: 0.70 vs 0.65; *P* = .02) or ECV (AUC: 0.70 vs 0.65; *P* = .002) to ARVC risk score increased the capability for predicting the occurrence of sustained VA; and (*c*) combined assessment of cardiac MRI biventricular GLS and ECV provided complementary and incremental information in predicting VA beyond ARVC risk score in patients with ARVC (AUC: 0.73 vs 0.65; *P* < .001).

Our study revealed that RV GLS and LV GLS derived using cardiac MRI FT were associated with the risk of VA during follow-up in ARVC. Cardiac MRI FT has proven to be a reliable technique for the accurate assessment of myocardial deformation and yielded excellent intra- and interobserver reproducibility in the current study (8–11). Recent reports have demonstrated the diagnostic value of cardiac MRI FT for ARVC, and its prognostic role in ARVC has gradually gained attention as well (9,22,23). As shown by others and our group, reduced RV and LV strain derived from cardiac MRI FT were associated with an increased risk of VA during follow-up for ARVC (15,16). This finding is not unexpected because reduced RVEF and LVEF have been shown to increase the relative risk of VA in ARVC (6,24); however, LV strain impairment was observed in patients with preserved LVEF in our sample and is considered to occur before a decrease in LVEF (12), providing evidence that the myocardial strain may be a more sensitive prognostic marker of VA than LVEF (12). Notably, GLS showed a greater predictive potential than global circumferential peak strain and global radial peak strain in the univariable analysis. To our knowledge, the myocardial fibers with longitudinal alignment have the highest susceptibility to dysfunction in various cardiac abnormalities, which means that impairment in longitudinal function precedes a reduction in circumferential indexes, leading to subclinical impairment of ventricular pump function (25). In addition, because longitudinal motion makes a predominant contribution to ventricular output, the reduction in GLS appears to have an intense association with ventricular dysfunction (26). Indeed, previous studies have demonstrated the predictive value of GLS in various cardiovascular diseases, indicating that GLS may be an appropriate parameter for global cardiac function evaluation and prognostication (13,14). These results indicate that cardiac MRI FT has potential to be a promising clinical tool, owing to its ability to monitor subtle changes in myocardial deformation in patients with ARVC during serial examinations.

In this study, we showed that biventricular GLS could provide incremental information beyond the ARVC risk score. This is noteworthy because more recent studies have shown that most cases of ARVC involve biventricular changes (3,4), whereas the final ARVC risk score did not consider LV involvement in the disease process, which seemed to underestimate the risk of patients with ARVC having LV involvement (3,27). Our finding of the incremental value of biventricular global strain underlines the fact that ARVC is a disease that affects not only RV but also LV. Although a recent study found no significant additional benefit of biventricular strain for prognosis (16), the diverging results may be explained by different methods and heterogeneous study populations, in which study participants with LV involvement were in different percentages in the two groups. In our study sample, more than half of the patients with ARVC exhibited evidence of LV involvement, including a part of patients with preserved LVEF but impaired global strain. Moreover, the proportion of patients with reduced LVEF in our study is higher than in previous studies (3). Besides including more patients with LV involvement, another possible explanation could be that a significant subset of patients in our study were hospitalized individuals with severe phenotypes. Furthermore, Aquaro et al (3) also demonstrated the prognostic utility of cardiac MRI in ARVC, and they found LV involvement at cardiac MRI was associated with an increased risk of VA in patients with ARVC, which is of particular concern. Therefore, biventricular features should be appropriately evaluated in patients with ARVC, and adding biventricular strain to the ARVC risk score may help in screening for patients with a higher risk of VA.

We found that ECV was associated with the risk of VA and provided incremental information beyond the ARVC risk score for predicting sustained VA in ARVC. To our knowledge, LV histologic involvement, specifically the presence of fibrofatty tissue deposits in the extracellular interstitium, has been frequently reported in explanted hearts or autopsy cases of ARVC, even in the absence of macroscopic affliction (28); this underlines the hypothesis of potential LV affliction in ARVC from an early stage (29). Excessive fibrosis contributes to ventricular stiffness and dysfunction and provides a substrate for re-entrant arrhythmias, resulting in increased VA and SCD (1). ECV, derived using T1 mapping technique, has been proven as the most correlated cardiac MRI index associated with histopathologic diffuse myocardial fibrosis (30). A higher ECV represents increasing myocardial fibrosis, and as anticipated, our study found that it was associated with a higher incidence of sustained VA. Moreover, the prognostic value of ECV for cardiac outcomes has been validated in various cardiac diseases (31,32). In contrast, native T1 did not show a significant association with sustained VA in ARVC. This can be explained by the fact native T1 is a measure of both intracellular and extracellular compartments of the myocardium and can be influenced by various factors including cardiomyocytes, hematocrit, and magnetic field strength (32,33). These factors might diminish the prognostic utility of native T1 compared with ECV. Conversely, ECV, normalized by the change in blood T1 relaxation rate between pre- and postcontrast imaging, is less sensitive to interindividual variability and field strength (32,33). Considering the aforementioned findings, ECV has potential to serve as a valuable supplementary biomarker in the current ARVC risk model for improved risk stratification.

Intriguingly, our findings indicated that combined biventricular GLS and ECV assessment improved risk stratification in ARVC to the greatest extent. A recent study suggested that ECV and GLS, evaluating respectively tissue characteristics of diffuse myocardial fibrosis and subtle contractile dysfunction, may represent principal but distinct metrics of cardiac vulnerability to adverse events, potentially reflecting distinct cellular origins (34). More precisely, ECV may indicate architectural distortion, most often resulting from diffuse myocardial fibrosis mediated by fibroblasts (35), whereas GLS may indicate cardiomyocyte contractile dysfunction. Each of them may reflect the fundamental myocardial derangements arising from distinct cell types. Owing to the weak correlation between ECV and GLS, combining both ECV and GLS may optimize the pathophysiologic understanding of tissue characteristics and function in cardiac disease, thereby being beneficial for the development of treatment strategies (34). Our preliminary exploration may fuel relevant studies, and more evidence from larger prospective studies is warranted to support or verify our findings and apply them in clinical practice.

Our study had several limitations. First, it was retrospective with a limited sample size. Second, although the findings suggest that cardiac MRI biventricular GLS and ECV were associated with sustained VA in ARVC, the results of this study should be considered hypothesis-generating and exploratory, and prospective validation is needed before recommending their widespread clinical application. Furthermore, the exclusion of 19 patients due to uninterpretable image quality might have affected patient selection and event stratification. In addition, in a portion of our population, cardiac MRI examination was indicated to monitor the progression of ARVC instead of disease detection, suggesting that these patients might have been in a relatively advanced stage of ARVC with a severe phenotype. Consequently, our findings may not be universally extrapolated to patients in early disease stages. Finally, the systematic investigation of other potential prognostic markers for ARVC is warranted.

In conclusion, biventricular GLS and LV ECV provided independent and incremental information for predicting sustained VA beyond the established ARVC risk score in patients with ARVC. Consequently, assessing GLS and ECV might contribute to risk stratification in ARVC. However, the results of this exploratory analysis should be validated by future studies.
